# Supplementary material for: Allosteric regulation and crystallographic fragment screening of SARS-CoV-2 NSP15 endoribonuclease
Source: Nucleic Acids Res. 2023 Apr 28;51(10):5255–70. doi: 10.1093/nar/gkad314 (PMC10250223; doi:10.1093/nar/gkad314)
Supplement: gkad314_Supplemental_Files [file gkad314_supplemental_files.zip › Supplementary datasets.zip!/188/Refine_9/01-BUSTER/Cycle-2/shell.01/R_Rfree.html]

# Rxpct factors in resolution bins at cycle 0


|  |  |  |  |  |  |  |  |  |
| --- | --- | --- | --- | --- | --- | --- | --- | --- |
|  | **<d> (Å)**  Rxpct\_Work | Rxpct\_Free | Rxpct\_All | |  |  |  |  |
|  | 16.0655 | 0.3886 | 0.5086 | 0.3926 |  |
|  | 9.2754 | 0.2209 | 0.2733 | 0.2240 |  |
|  | 7.1847 | 0.1972 | 0.2352 | 0.1993 |  |
|  | 6.0722 | 0.2007 | 0.2577 | 0.2032 |  |
|  | 5.3552 | 0.1627 | 0.2770 | 0.1674 |  |
|  | 4.8439 | 0.1513 | 0.2090 | 0.1547 |  |
|  | 4.4558 | 0.1506 | 0.2160 | 0.1540 |  |
|  | 4.1481 | 0.1551 | 0.2234 | 0.1591 |  |
|  | 3.8965 | 0.1654 | 0.2289 | 0.1684 |  |
|  | 3.6857 | 0.1729 | 0.2483 | 0.1762 |  |
|  | 3.5058 | 0.1756 | 0.2185 | 0.1777 |  |
|  | 3.3499 | 0.1790 | 0.2383 | 0.1823 |  |
|  | 3.2131 | 0.1906 | 0.2161 | 0.1921 |  |
|  | 3.0918 | 0.1917 | 0.2356 | 0.1937 |  |
|  | 2.9833 | 0.1919 | 0.2228 | 0.1932 |  |
|  | 2.8855 | 0.2045 | 0.2385 | 0.2062 |  |
|  | 2.7967 | 0.2056 | 0.2404 | 0.2071 |  |
|  | 2.7156 | 0.2091 | 0.2408 | 0.2106 |  |
|  | 2.6412 | 0.2623 | 0.2677 | 0.2627 |  |
|  | 2.5725 | 0.3549 | 0.2805 | 0.3524 |  |
|  | **Overall** | 0.1889 | 0.2428 | 0.1916 |  |

# Rxpct factors in resolution bins at cycle 1


|  |  |  |  |  |  |  |  |  |
| --- | --- | --- | --- | --- | --- | --- | --- | --- |
|  | **<d> (Å)**  Rxpct\_Work | Rxpct\_Free | Rxpct\_All | |  |  |  |  |
|  | 16.0655 | 0.3891 | 0.5085 | 0.3931 |  |
|  | 9.2754 | 0.2210 | 0.2737 | 0.2241 |  |
|  | 7.1847 | 0.1975 | 0.2354 | 0.1996 |  |
|  | 6.0722 | 0.2011 | 0.2582 | 0.2036 |  |
|  | 5.3552 | 0.1625 | 0.2771 | 0.1672 |  |
|  | 4.8439 | 0.1511 | 0.2092 | 0.1545 |  |
|  | 4.4558 | 0.1507 | 0.2163 | 0.1541 |  |
|  | 4.1481 | 0.1550 | 0.2227 | 0.1589 |  |
|  | 3.8965 | 0.1650 | 0.2293 | 0.1680 |  |
|  | 3.6857 | 0.1725 | 0.2496 | 0.1759 |  |
|  | 3.5058 | 0.1754 | 0.2181 | 0.1775 |  |
|  | 3.3499 | 0.1787 | 0.2380 | 0.1821 |  |
|  | 3.2131 | 0.1905 | 0.2154 | 0.1920 |  |
|  | 3.0918 | 0.1917 | 0.2357 | 0.1936 |  |
|  | 2.9833 | 0.1917 | 0.2226 | 0.1930 |  |
|  | 2.8855 | 0.2042 | 0.2378 | 0.2059 |  |
|  | 2.7967 | 0.2054 | 0.2406 | 0.2069 |  |
|  | 2.7156 | 0.2088 | 0.2402 | 0.2103 |  |
|  | 2.6412 | 0.2618 | 0.2678 | 0.2622 |  |
|  | 2.5725 | 0.3536 | 0.2808 | 0.3512 |  |
|  | **Overall** | 0.1888 | 0.2428 | 0.1916 |  |

# Rxpct factors in resolution bins at cycle 2


|  |  |  |  |  |  |  |  |  |
| --- | --- | --- | --- | --- | --- | --- | --- | --- |
|  | **<d> (Å)**  Rxpct\_Work | Rxpct\_Free | Rxpct\_All | |  |  |  |  |
|  | 16.0655 | 0.3892 | 0.5085 | 0.3931 |  |
|  | 9.2754 | 0.2211 | 0.2737 | 0.2242 |  |
|  | 7.1847 | 0.1975 | 0.2355 | 0.1997 |  |
|  | 6.0722 | 0.2010 | 0.2581 | 0.2035 |  |
|  | 5.3552 | 0.1625 | 0.2773 | 0.1672 |  |
|  | 4.8439 | 0.1511 | 0.2092 | 0.1545 |  |
|  | 4.4558 | 0.1506 | 0.2161 | 0.1540 |  |
|  | 4.1481 | 0.1549 | 0.2228 | 0.1588 |  |
|  | 3.8965 | 0.1649 | 0.2295 | 0.1680 |  |
|  | 3.6857 | 0.1725 | 0.2494 | 0.1759 |  |
|  | 3.5058 | 0.1754 | 0.2180 | 0.1775 |  |
|  | 3.3499 | 0.1787 | 0.2380 | 0.1821 |  |
|  | 3.2131 | 0.1906 | 0.2153 | 0.1920 |  |
|  | 3.0918 | 0.1917 | 0.2359 | 0.1937 |  |
|  | 2.9833 | 0.1917 | 0.2227 | 0.1930 |  |
|  | 2.8855 | 0.2042 | 0.2377 | 0.2059 |  |
|  | 2.7967 | 0.2054 | 0.2405 | 0.2069 |  |
|  | 2.7156 | 0.2088 | 0.2402 | 0.2103 |  |
|  | 2.6412 | 0.2617 | 0.2671 | 0.2621 |  |
|  | 2.5725 | 0.3536 | 0.2788 | 0.3511 |  |
|  | **Overall** | 0.1888 | 0.2428 | 0.1915 |  |

# Rxpct factors in resolution bins at cycle 3


|  |  |  |  |  |  |  |  |  |
| --- | --- | --- | --- | --- | --- | --- | --- | --- |
|  | **<d> (Å)**  Rxpct\_Work | Rxpct\_Free | Rxpct\_All | |  |  |  |  |
|  | 16.0655 | 0.3851 | 0.5102 | 0.3892 |  |
|  | 9.2754 | 0.2221 | 0.2791 | 0.2255 |  |
|  | 7.1847 | 0.1973 | 0.2368 | 0.1996 |  |
|  | 6.0722 | 0.1997 | 0.2469 | 0.2017 |  |
|  | 5.3552 | 0.1592 | 0.2711 | 0.1638 |  |
|  | 4.8439 | 0.1487 | 0.2159 | 0.1526 |  |
|  | 4.4558 | 0.1450 | 0.2062 | 0.1482 |  |
|  | 4.1481 | 0.1507 | 0.2051 | 0.1538 |  |
|  | 3.8965 | 0.1573 | 0.2287 | 0.1607 |  |
|  | 3.6857 | 0.1674 | 0.2530 | 0.1713 |  |
|  | 3.5058 | 0.1716 | 0.2077 | 0.1734 |  |
|  | 3.3499 | 0.1787 | 0.2358 | 0.1820 |  |
|  | 3.2131 | 0.1915 | 0.2159 | 0.1929 |  |
|  | 3.0918 | 0.1916 | 0.2494 | 0.1942 |  |
|  | 2.9833 | 0.1941 | 0.2403 | 0.1961 |  |
|  | 2.8855 | 0.2054 | 0.2431 | 0.2073 |  |
|  | 2.7967 | 0.2056 | 0.2518 | 0.2076 |  |
|  | 2.7156 | 0.2109 | 0.2345 | 0.2121 |  |
|  | 2.6412 | 0.2673 | 0.2615 | 0.2669 |  |
|  | 2.5725 | 0.3542 | 0.2690 | 0.3514 |  |
|  | **Overall** | 0.1866 | 0.2414 | 0.1893 |  |
